# Rhombohedral boron monosulfide as a metal-free photocatalyst

**DOI:** 10.1038/s41598-023-46769-7

**Published:** 2023-11-09

**Authors:** Keisuke Miyazaki, Akira Yamaguchi, Haruki Kusaka, Norinobu Watanabe, Aufandra Cakra Wardhana, Satoshi Ishii, Akiyasu Yamamoto, Masashi Miyakawa, Takashi Taniguchi, Takahiro Kondo, Masahiro Miyauchi

**Affiliations:** 1 Department of Materials Science and Engineering, School of Materials and Chemical Technology, Tokyo Institute of Technology, Meguro‐ku, Tokyo, 152‐8552 Japan; 2 Department of Materials Science, Institute of Pure and Applied Sciences, University of Tsukuba, Tsukuba, 305‐8573 Japan; 3 Research Center for Materials Nanoarchitectonics, National Institute for Materials Science, Tsukuba, 305‐0044 Japan; 4 Institute of Engineering, Tokyo University of Agriculture and Technology, Tokyo, 183-8538 Japan; 5 The Advanced Institute for Materials Research, Tohoku University, 2-1-1 Sendai, Miyagi, 980-8577 Japan; 6 Tsukuba Research Center for Energy Materials Science, Institute of Pure and Applied Sciences and R&D Center for Zero CO2 Emission Functional Materials, University of Tsukuba, Tsukuba, 305‐8573 Japan

**Keywords:** Photocatalysis, Photocatalysis

## Abstract

Most of previous photocatalysts contain metal species, thus exploring a metal-free photocatalyst is still challenging. A metal-free photocatalyst has an advantage for the development of economical and non-toxic artificial photosynthesis system and/or environmental purification applications. In this study, rhombohedral boron monosulfide (r-BS) was synthesized by a high-pressure solid-state reaction, and its photocatalytic properties were investigated. r-BS absorbed visible light, and its photocurrent action spectrum also exhibited visible light responsivity. The r-BS evolved hydrogen (H_2_) from water under ultraviolet (UV) as well as under visible light irradiation, and its internal quantum efficiency reached 1.8% under UV light irradiation. In addition to the H_2_ evolution reaction, the r-BS photocatalyst drove carbon dioxide (CO_2_) reduction and dye oxidation reactions under UV irradiation. Although bare r-BS was not so stable under strong light irradiation in water, cocatalyst modification improved its stability. These results indicate that r-BS is a new class of non-metal photocatalyst applicable for H_2_ production, CO_2_ reduction, and environmental purification reactions.

## Introduction

Since the discovery of photoelectrochemical water splitting by titanium dioxide (TiO_2_)^1^, various semiconductor-based photocatalysts, including metal oxides and metal sulfides, have been reported^2, 3^. Most of these photocatalysts contain metal species, which can sometimes pose disadvantages for economical and/or non-toxic photocatalysis applications. Thus, exploring a metal-free photocatalyst is challenging for the development of an economical and safe artificial photosynthesis system and/or an environmental purification system.

As a metal-free photocatalyst, Maeda et.al. reported graphitic carbon nitride (g-C_3_N_4_)^4, 5^. g-C_3_N_4_ is a narrow bandgap semiconductor; thus, many groups have examined this material as a visible-light-responsive photocatalyst for water splitting^6–12^. In addition to g-C_3_N_4_, boron-based materials attract great attention^13^. For example, boron oxide (B_6_O)^14^, boron phosphide (BP)^15^, boron-based hydride^16^, hexagonal boron nitride (h-BN)^17–19^, boron carbonitride (BCN)^20, 21^, and h-BN/graphene hybrid^22^ have been reported. The reports of non-metal photocatalysts are limited at present, and thus exploring a new class of metal-free photocatalysts is still a challenging issue.

Herein, we report rhombohedral boron monosulfide (r-BS) as an active metal-free photocatalyst. The synthesis of r-BS has previously been achieved by a high-pressure solid-state reaction (5.5 GPa, 1600 °C)^23–26^. Although its superconducting properties^27^, thermoelectric properties^28^, hydrogen storage capacities^29^, and electrocatalytic properties^30^ have been theoretically examined, its photo-electrochemical and photocatalytic properties have not yet been studied. Recently, Kusaka et al. experimentally studied the tunable bandgap of r-BS by its morphological change^24^, thus it is expected to act as a visible-light-sensitive photocatalyst. In the present study, the photocatalytic properties of r-BS are comprehensively evaluated, including hydrogen (H_2_) production, carbon dioxide (CO_2_) reduction, and methylene blue (MB) dye oxidation. Furthermore, we discuss the visible light sensitivity and stability of r-BS.

## Results

### Characterization

**Figure 1:**
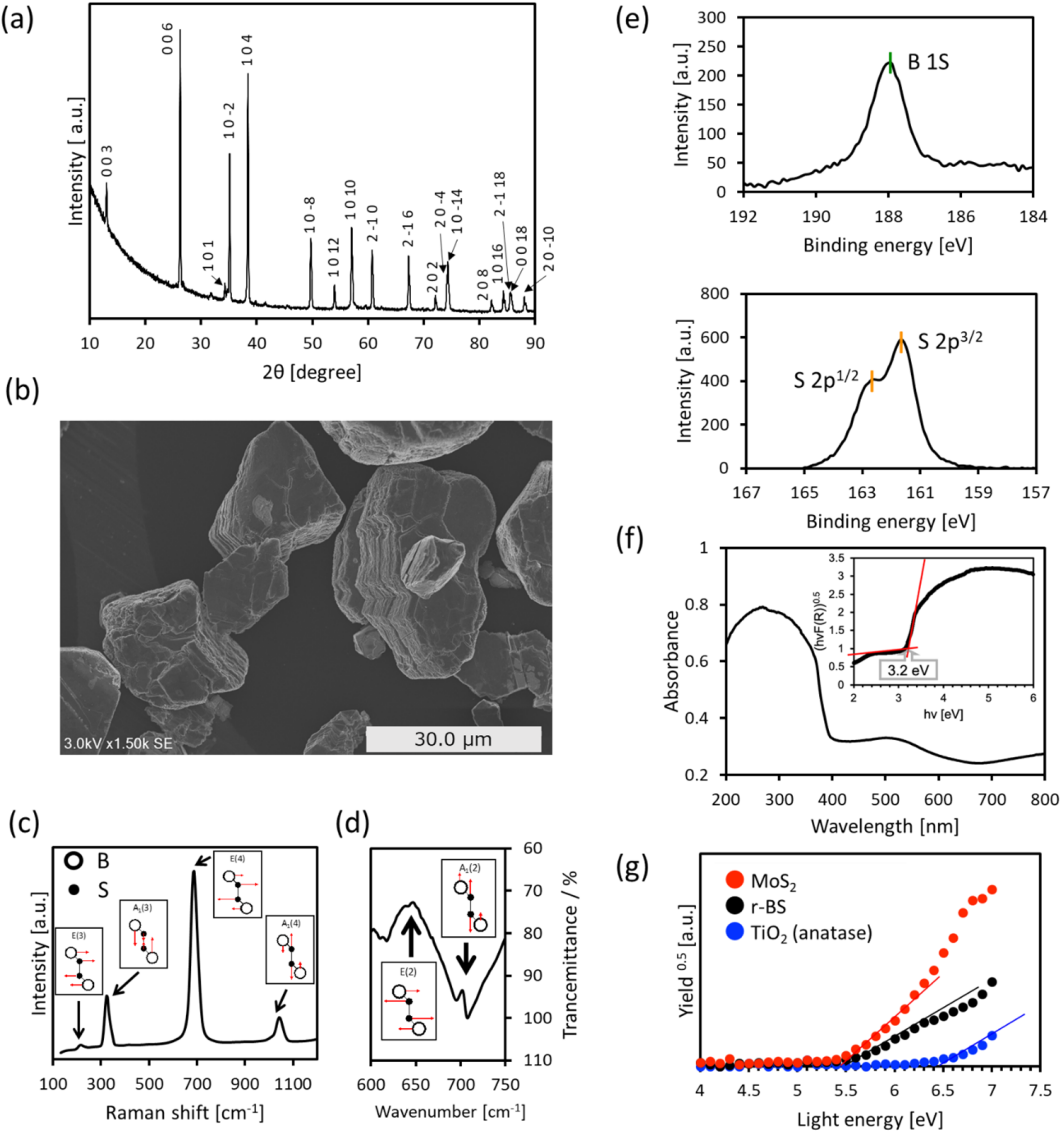
(**a**) X-ray diffraction (XRD) pattern of r-BS powder. (**b**) Field emission scanning electron microscope (FE-SEM) image of r-BS powder. (**c**) Raman spectrum of r-BS powder (insert figures show the schematic illustration of vibration modes). (**d**) Fourier Transform Infrared Spectroscopy (FT-IR) of r-BS powder (insert figures show the schematic illustration of vibration modes). (**e**) X-ray photoelectron spectroscopy (XPS) signals of B 1s and S 2p of powder r-BS. (**f**) Optical UV–Vis absorption spectrum of r-BS powder (insert figure shows its Tauc plot). (**g**) Spectra of photoemission yield spectroscopy (PYS) of r-BS powder (black circles), MoS_2_ (red circles) and TiO_2_ (blue circles).

The r-BS powder was synthesized by a previously reported high-pressure solid-state reaction^24^. Figure 1a shows the X-ray diffraction (XRD) pattern of the r-BS powder. All peaks were assigned to the trigonal r-BS with the $R\overline{3 }m$ space group symmetry^24^. Figure 1b shows the field emission scanning electron microscope (FE-SEM) image of the r-BS powder. The morphology of r-BS suggests a layered structure, which is consistent with the layered crystal structure of r-BS^23^. The previous paper reported that the r-BS nanosheets were exfoliated by a scotch-tape method or dispersion in acetonitrile^24^, while most of the r-BS particles crushed in a mortar were three-dimensional bulk. In the present paper, we used r-BS powder just after being crushed in an agate mortar for characterization and evaluations, thus the present r-BS particles exist with a three-dimensional bulk nature. As shown in Fig. 1c, the Raman spectrum of r-BS exhibits four peaks. The peaks at 210 cm^−1^, 320 cm^−1^, 690 cm^−1^, and 1050 cm^−1^ are assigned to the E(3), A_1_(4), E(4), and A_1_(4) modes of r-BS, respectively. The schematic explanation of these modes in Raman spectra is shown in Supporting Information (Fig. S1)^25^. We also measured Fourier transform infrared spectroscopy (FT-IR) spectrum (Fig. 1d). In the wavenumber range of 600 cm^−1^ to 750 cm^−1^, a relatively large mountain-valley shape was observed. According to the previous report^31^, mountain-valley shape is originated from the Fano resonance between the E(2) (TO) phonon mode and charge carriers. At the wavenumber of 700 cm^−1^, the small peak with mountain shape was observed. This peak is assigned to the A_1_(2) (TO) phonon mode^25^. The wide-scanned result of X-ray photoelectron spectroscopy (XPS) for r-BS put on a graphite tape is shown in Supporting Information (Fig. S2). Besides the signals from a graphite tape, all peaks were assigned to boron or sulfur orbitals. Figure 1e shows the narrow-scanned XPS spectra for B-1s and S-2p orbitals. It is clearly seen that the S-2p signal has doublet peaks for S-2p_3/2_ and S-2p_1/2_ core levels. Based on the peak positions, the charge states of B and S were almost neutral, similar to the previous report^24^. We previously investigated the chemical composition and stability of r-BS by XPS^24^. The B : S atomic ratio was calculated to be 1 ± 0.1:1 ± 0.1, and r-BS was stable under air and water droplet exposure. Figure 1f shows the optical ultraviolet- visible (UV–Vis) absorption spectrum of r-BS. Two-step optical absorption was seen, i.e. strong absorption below 400 nm and broad absorption around 400–600 nm. The previous study performed DFT calculation on r-BS, suggesting that r-BS is an indirect semiconductor^24^. Thus, the Tauc plot (inset of Fig. 1f) uses the square root of absorption as a vertical axis. Then, the optical bandgap determined by strong absorption in the Tauc plot was approximately 3.2 eV. Previously, Sasaki et al. reported the UV–Vis spectrum of r-BS, which also exhibited two steps of optical absorption^23^. According to this report, the strong absorption that appeared in the ultraviolet (UV) region was assigned to the bandgap excitation, while the weak absorption in the visible light range would be originated from some deficiencies in r-BS. The results of photoemission yield spectroscopy (PYS) are shown in Fig. 1g, which are useful for discussing the position of the valence band maximum (VBM) of semiconductors^32–34^. The present PYS analysis was not conducted in a vacuum; therefore, we could not relate it directly to the vacuum energy level. Hence, we referred to the data of titanium dioxide (TiO_2_) with anatase phase as a well-known photocatalyst and molybdenum disulfide (MoS_2_) as a well-studied metal sulfide. As shown in the PYS spectra, the VBM position of r-BS was approximately 1.0 V shallower than that of TiO_2_ and close to that of MoS_2_. While some reports indicate that the VBM of MoS_2_ is located at a more positive potential than the redox potential of oxygen (O_2_) evolution from water^35–37^, reports of photocatalytic water oxidation by MoS_2_ are still limited because of the large overpotential for water oxidation. These previous studies imply that water oxidation by r-BS is challenging, as will be discussed later. In contrast, the position of the conduction band minimum (CBM) of r-BS is negative enough to drive H_2_ production, as its bandgap value was 3.2 eV as shown in Fig. 1f. The bandgap value and the position of the valence band of r-BS affect the photocatalytic property of r-BS as described below.

### Photoelectrochemical property

**Figure 2:**
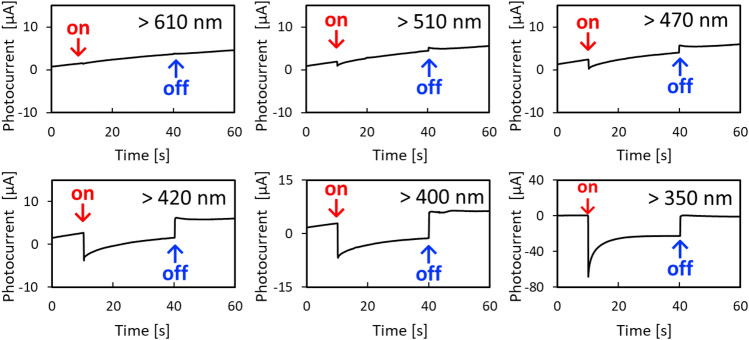
Photoelectrochemical current response under light on and off condition (Light irradiation from 10 to 40 s). Light was irradiated using a xenon lamp through short wavelength cut-off filter below 610 nm, 510 nm, 470 nm, 420 nm, 400 nm, 350 nm, respectively.

We evaluated the wavelength dependence of the photoelectrochemical properties of an r-BS electrode to determine its responsive photon energy. Figure 2 shows the changes in the photocurrent of the r-BS electrode for various wavelengths using a xenon lamp with short-wavelength cutoff filters (Fig. S11a in Supporting Information) under the rest potential condition. A negative photocurrent was observed in each case, indicating the p-type nature of the r-BS^38^. Although the photocurrent densities under UV light were more obvious, those under visible light shorter than 510 nm were still responsive. These results imply that two-step absorption under UV and visible light shown in Fig. 1f are responsive to the photocurrent generation. The linear sweep voltammogram of the r-BS electrode under chopping light irradiation by a 150 W xenon lamp is also shown in Supporting Information (Fig. S3). The significant photocurrent under cathodic bias also implies the visible-light sensitivity and the p-type nature of r-BS.

### Hydrogen evolution

**Figure 3:**
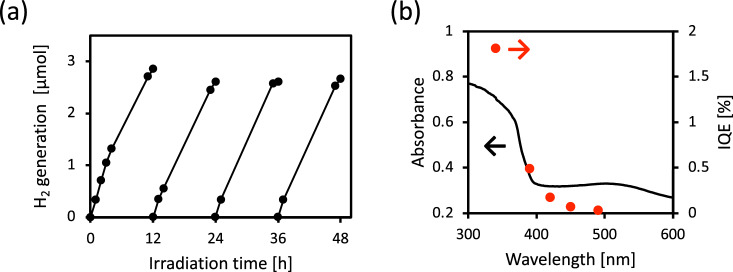
(**a**) Time course of H_2_ evolution from water containing ethanol as sacrificial agent under a xenon lamp. The head space of reactor was replaced with Ar gas every 12 h. (**b**) Black line is the optical absorbance of r-BS and red dots are *IQE* of r-BS for hydrogen generation at the different wavelengths light.

Next, we evaluated the H_2_ evolution property of r-BS powder dispersed in water with the addition of ethanol as a sacrificial agent under xenon lamp irradiation. Figure 3a shows the repeated H_2_ production property of r-BS after the evacuation of the headspace gas in a reactor for each measurement. H_2_ molecules were produced four times at a constant rate, each H_2_ production session lasted 12 h. Furthermore, we examined the action spectrum of the internal quantum efficiency (*IQE*) for H_2_ generation by the r-BS photocatalyst under various wavelength conditions (The *IQE* calculation procedure is described in Supporting Information, Note 1). As shown in Fig. 3b, H_2_ molecules were generated even under visible light up to 500 nm and were generated under UV irradiation, reaching approximately 1.8% of *IQE*. The limited *IQE* under visible light would originate from defects in r-BS, as reported by Sasaki et al.^23^. During the H_2_ production reaction shown in Fig. 3, ethanol would be oxidized under light irradiation conditions. O_2_ production was barely detected in the absence of sacrificial agents. These results indicate that it is difficult for r-BS to produce O_2_ from water; however, as mentioned later, we confirmed photocatalytic dye oxidation by r-BS. These results indicate that r-BS can be used in potential applications, such as p-type photocathodes for H_2_ production and environmental purification of harmful organic chemical compounds.

### Carbon dioxide reduction

**Figure 4:**
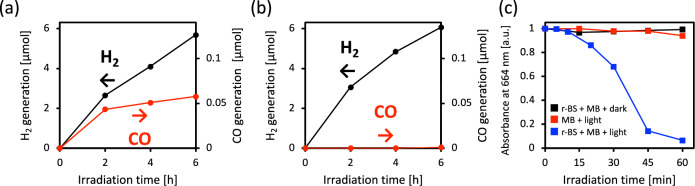
(**a**) The generated amount of CO (red line) and H_2_ (black line) by r-BS under a xenon lamp irradiation in the CO_2_ bubbled condition with a TEOA sacrificial agent. (**b**) Shows the amount of CO (red line) and H_2_ (black line) by r-BS under a Xe lamp irradiation in the Ar bubbled condition with a TEOA sacrificial agent. (**c**) Shows MB dye decomposition by r-BS under UV irradiation.

In addition to the H_2_ evolution reaction, the photocatalytic carbon dioxide (CO_2_) reduction by r-BS was evaluated. The r-BS powder was dispersed in an aqueous solution containing triethanolamine (TEOA) as a sacrificial agent for the oxidation reaction. We used TEOA instead of ethanol for CO_2_ reduction because ethanol is oxidized to CO_2_. We compared the products generated under CO_2_ bubbled and argon (Ar) bubbled conditions under the same xenon lamp irradiation conditions shown in Fig. 3a. Figure 4 shows the results under CO_2_ bubbling (a) and Ar bubbling (b). It is noteworthy that r-BS generated carbon monoxide (CO) under the CO_2_ bubbled condition, while no CO was detected under the Ar bubbling condition. These results indicated that r-BS can drive the photocatalytic CO_2_ reduction to CO. To verify whether the detected CO originated from the reduction of bubbled CO_2_, we conducted an isotope trace experiment that involved gas chromatography-mass spectroscopy (GC–MS) using isotope CO_2_ with a carbon mass number of 13 (^13^CO_2_) for bubbling. The peak of CO with a mass number of 29 (^13^CO) appears at approximately 2.5 min retention time in a GC–MS chart (Fig. S4 in Supporting Information). As a result, ^13^CO was not detected under an ordinal carbon dioxide (^12^CO_2_) atmosphere, whereas ^13^CO was detected under ^13^CO_2_ conditions. These isotope analyses also clearly reveal the photocatalytic CO_2_ reduction ability of r-BS.

### Dye decomposition

Next, we evaluated the photocatalytic dye degradation by an oxidation reaction, since it is useful to discuss the capability of r-BS for environmental remediation applications, such as water purification, antibacterial functions, and antiviral functions. In this study, methylene blue (MB) dye molecules were used for photocatalysis tests in aqueous media. Figure 4c shows the change in absorbance values of MB under Hg-Xe lamp irradiation passed through a 360 nm bandpass filter, which mainly excites r-BS to avoid the excitation of MB itself. We measured the change in the absorbance of MB under dark conditions to check MB adsorption onto r-BS; the absorbance of MB did not decrease significantly under dark conditions (black squares in Fig. 4c). Furthermore, only MB without r-BS under light irradiation exhibited any changes in its absorbance values (red squares in Fig. 4c), indicating that the photolysis of MB was limited under the present experimental conditions. In particular, MB with r-BS under light irradiation caused the decoloration of the MB dye. It is known that the MB molecule turns transparent by both oxidation and reduction reactions, but the reduced MB with the Leuco form turned blue in the dark under an oxygen-rich condition^39^. To check if the present MB decoloration is driven by the oxidation or reduction reaction, we investigated the recovery of the absorbance values of MB under O_2_ bubbling conditions after light irradiation. As a result, the recovery of the absorbance values of MB was very limited (Fig. S5 in Supporting Information), indicating that oxidation is the dominant reaction in the MB decoloration process. These results reveal that r-BS can photocatalytically oxidize organic molecules and is useful in environmental remediation applications.

### Photo-stability test

Although constant H_2_ evolution was observed in Fig. 3a, metal sulfides are generally unstable owing to their self-oxidation reaction under light irradiation in aqueous media. Therefore, we conducted a durability test of r-BS under stronger light irradiation than that shown in Fig. 3a. The H_2_ production performance of r-BS decreased under strong light irradiation and remained constant after 4 h (Fig. S6 in Supporting Information). These results indicate that r-BS was partially photo-corroded. A comparison of the UV–Vis spectra and the XRD patterns of the r-BS samples before and after sufficient light irradiation (Fig. S7 in Supporting Information) reveals no significant changes. However, the H_2_ production performance deterioration, as shown in Fig. S6, suggests that r-BS is photo-corroded, similar to the previous metal sulfide cases^40, 41^. To check the morphology change of r-BS after light irradiation, we conducted the SEM observation of before and after photocatalytic reaction. As shown in Fig. S8a, pristine r-BS was layered structure with clear edge, however, the edge part of r-BS was broken or melted after light irradiation in water. We also conducted the XPS analysis on r-BS before and after the photocatalysis test (Fig. S9). After light irradiation onto r-BS, both binding energies of B-1s and S-2p orbitals were shifted towards smaller values, indicating the negatively charged state of r-BS after light irradiation.

**Figure 5:**
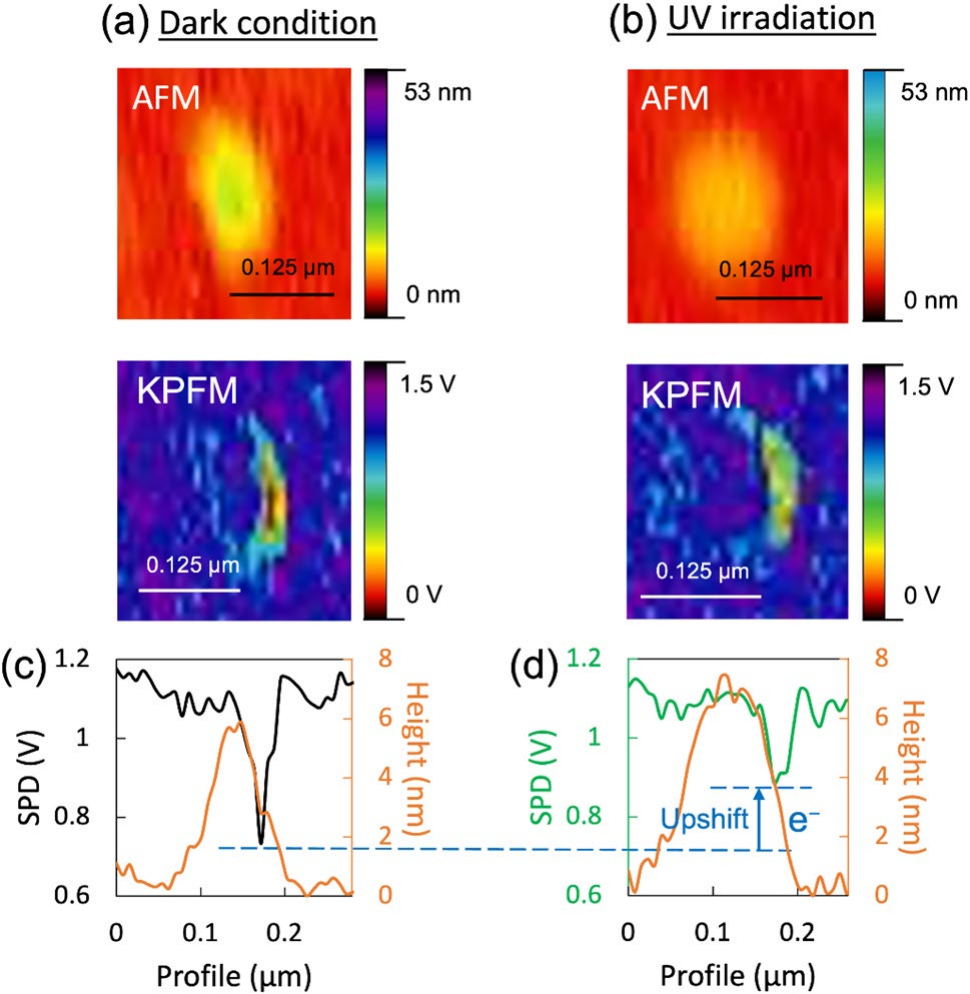
AFM and KPFM analysis of the same r-BS particle in the dark (**a**) and under UV irradiation (**b**). The average cross-sectional profiles of AFM and KPFM (**c** and **d**) were taken in vertical direction.

To investigate the charge separation in r-BS particle under light irradiation more in detail, we did the atomic force microscope (AFM) and the Kelvin-probe force microscope (KPFM) measurement for the r-BS particle under dark and UV irradiation condition. As Wardhana et al. reported, KPFM is a powerful tool to determine the relative work function and carrier shift of a semiconductor^42^. Figure 5a shows the AFM and KPFM results under the dark condition. These images would capture a layered r-BS small particle with its facet. The KPFM result suggests that the right edge of r-BS sheet has lower surface potential difference (SPD) than its facet face as shown in Fig. 5c. This result indicates that the local work function of r-BS at the edge is different from that of the facet position. Furthermore, a KPFM measurement with UV irradiation was conducted to observe the charge separation between the surface and the edge (Fig. 5b). During the irradiation, the Fermi level of the right edge upshifted (Fig. 5d), indicating the electrons accumulation to the edge. Therefore, in the case of our r-BS, the excited electrons were transferred to the edge of r-BS leaving holes in planer facet sites.

**Figure 6:**
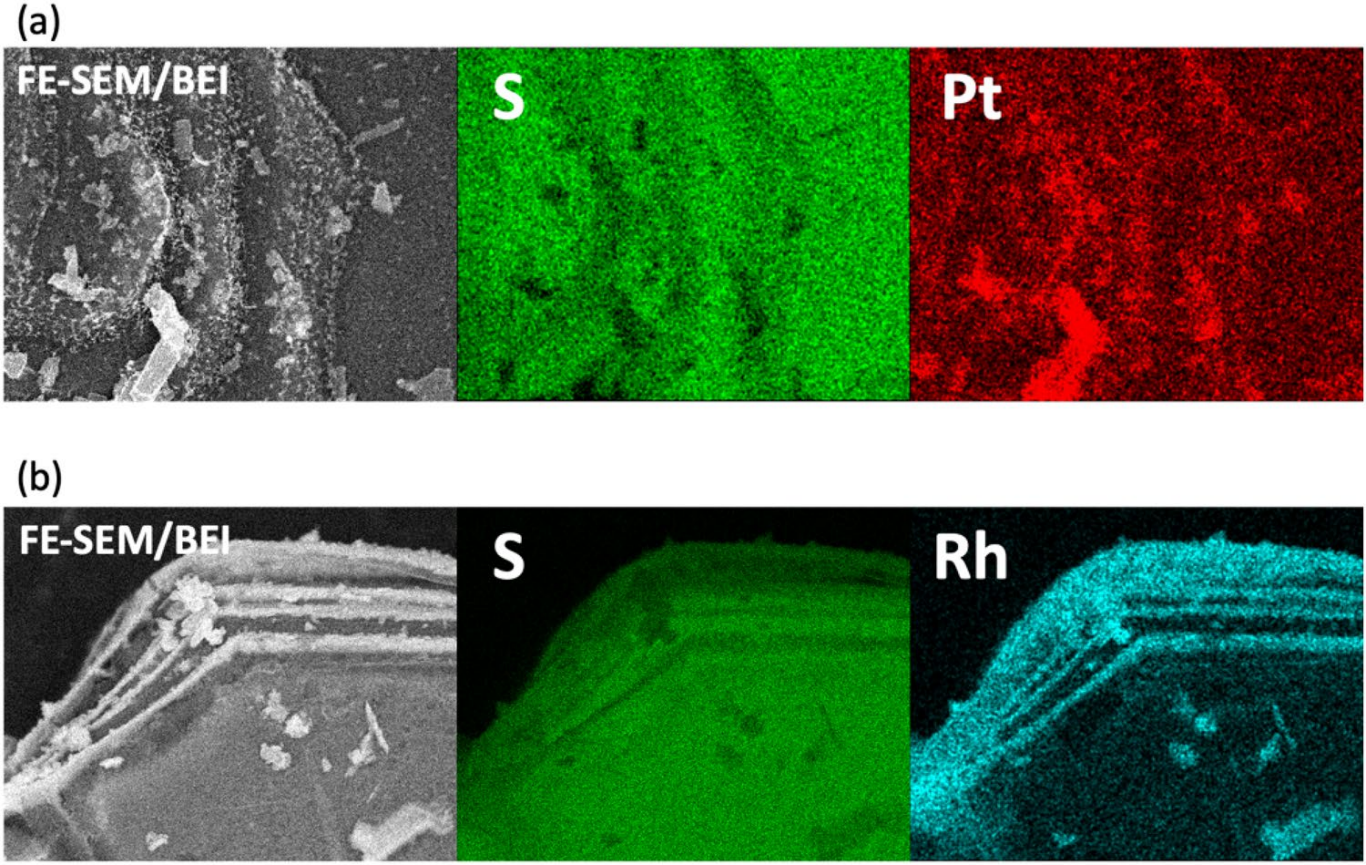
Backscattered electron image and EDS elemental mapping of Pt photo-deposited r-BS (**a**) and Rh photo-deposited r-BS (**b**).

In addition to the XPS and KPFM analyses, photocatalytic reduction or oxidation sites in r-BS were experimentally examined. So, we deposited platinum (Pt) or rhodium (Rh) nanoparticles onto r-BS by a photo-reduction method similar to the way of the reported literature^43^. Figure 6a and b show the SEM images and their EDS elemental mapping of Pt (a) and Rh loaded r-BS (b). Both Pt and Rh nanoparticles were mainly deposited on the edge of layered r-BS particles. These results indicate that the edge parts of r-BS act as photocatalytic reduction sites, consistent with the KPFM result. Based on these results, damaged r-BS shown in Fig. S8b is attributed to its photocatalytic self-reduction process similar to the previous cuprous oxide case^44^.

**Figure 7:**
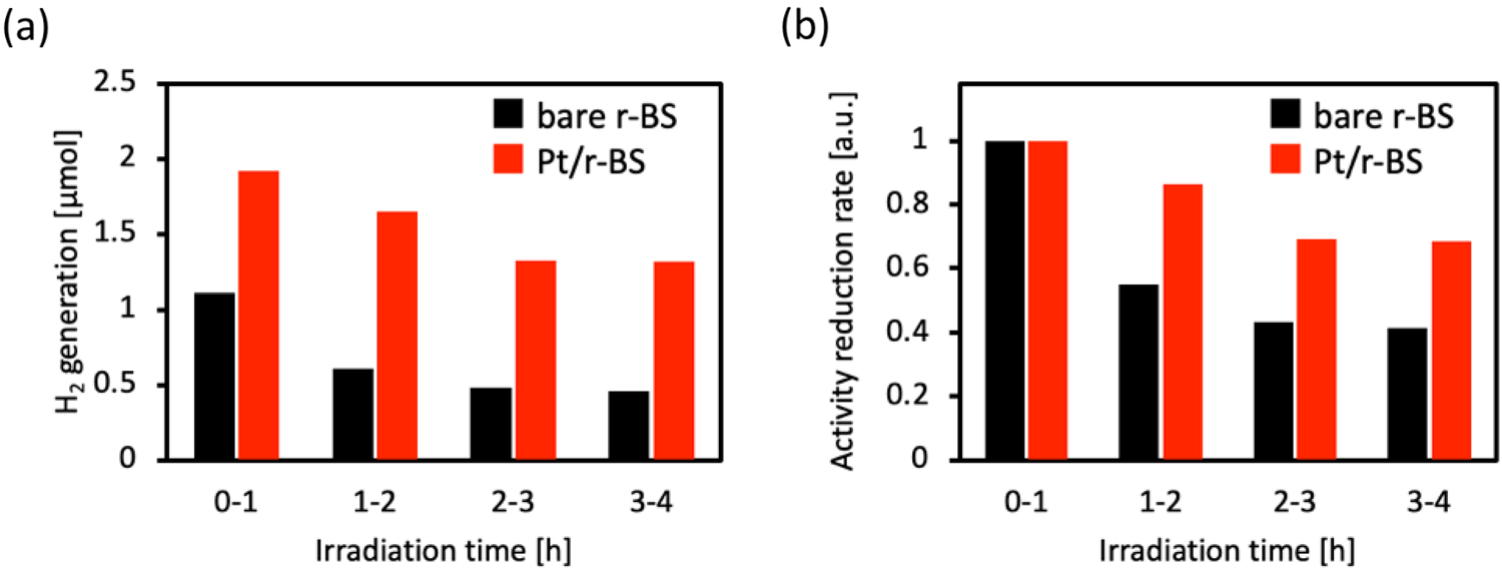
(**a**) H_2_ production amount under light irradiation for every hour of bare r-BS and Pt loaded r-BS. (**b**) Activity reduction rate calculated by normalizing the data in (**a**) at each 0-1 h H_2_ generation.

We expect that the Pt or Rh deposition improve the stability of r-BS, since their deposition inhibits the self-reduction of r-BS. Thus, we compared the photocatalytic H_2_ production activities and stabilities of the pristine r-BS with those of the Pt loaded r-BS. Figure 7a shows the raw data of H_2_ production for pristine r-BS and Pt-loaded r-BS. It is noted that the loading of Pt nanoparticles improves photocatalytic H_2_ production ability of r-BS by their cocatalyst effect. Figure 7b shows the normalized data of H_2_ production at the first 0–1 h irradiation, indicating that the Pt loading also improves the stability of r-BS during its photocatalytic H_2_ production. These results indicate that the loading of cocatalyst improves the stability of r-BS. In the present study, even though we used Pt as the most common cocatalyst for hydrogen production, other economical cocatalysts can be used to improve the reaction rate and the stability of an r-BS photocatalyst. We will evaluate the stability of both cocatalysts and r-BS in the future study.

## Conclusion

r-BS prepared under high-pressure conditions exhibited photocatalytic activities toward H_2_ production, CO_2_ reduction, and dye oxidation without any cocatalyst modification. It is responsive to visible light up to 500 nm in wavelength, and its *IQE* for H_2_ production under UV irradiation was 1.8%. Although the present r-BS was not perfectly stable under strong light-intensity conditions due to self-reduction reactions, a metal cocatalysts modification promoted the stability of r-BS. This study found that r-BS is a new class of non-metal photocatalysts. It consists of abundant elements and can potentially be used in economical artificial photosynthesis and/or environmental remediation systems.

## Methods

### r-BS synthesis

Amorphous boron was prepared by the decomposition of B_2_H_6_ and the obtained amorphous boron was mixed with sulfur powder (Wako Pure Chemical Industries Ltd., Osaka, Japan) in a 1:1 atomic ratio. Pellets of this mixture were formed by pressing; then, two pellets were placed in a capsule consisting of a boron nitride wall, sodium chloride (NaCl) disks, and graphite disks (Fig. S10 in Supporting Information). The pressure was first increased to 5.5 GPa, then, the temperature was increased to 1873 K over 17 min. After keeping the sample at 1873 K for 40 min, the temperature was reduced to room temperature (approximately 298 K). After release the pressure to ambient atmosphere, the r-BS pellet was carefully  taken out by removing the surrounding materials and crushed into a powder form for a photocatalyst test.

### Characterization

The crystal structure of r-BS was characterized using an X-ray diffractometer (Rigaku, Smart Lab, Tokyo, Japan) with CuKα radiation. A nonreflecting silicon plate was used as a sample stage, and the diffraction pattern was recorded using a D/TeX detector. The morphology of r-BS was observed using FE-SEM (Hitachi, Ltd., SU9000, Tokyo, Japan). The top of the valence band was estimated by photoelectron yield spectroscopy (RIKEN KEIKI Co., Ltd., AC-2, Tokyo, Japan). In the PYS analysis, an electron that was photoemitted from its valence band was accelerated away from the sample by a weak positive bias (sample was at 0 V) and in the process was picked up by an oxygen molecule in air. The singlet oxygen radical anion continued to accelerate away from the sample and subsequently entered a high voltage gradient chamber which caused an electron avalanche to occur through an electron cascading process involving nitrogen gas. The free electrons involved in this avalanche process were detected at the anode and through calibration were correlated to the photoelectron yield of the sample. Macroscopic charging of the sample was minimized by using very weak UV intensities (5–500 nW/cm^2^) for photoemission. The binding energies of B-1s and S-2p of r-BS were measured using X-ray photoelectron spectroscopy (JEOL Ltd., JPS 9010 TR, Tokyo, Japan) with an Mg Kα X-ray source, where the pass energy was set at 10 eV. The charge build-up in the sample (because of the incomplete contact of a graphite tape with a sample holder) resulted in a slight shift to higher binding energies for those spectra. Therefore, we calibrated the charge build-up based on the C-1s peak of a graphite tape at 284.6 eV. The Raman spectroscopy analysis of r-BS was performed using a Raman imaging system (JASCO Corporation, NRS-5100, Tokyo, Japan) with an incident light wavelength of 532 nm. Infrared spectra were measured using Fourier transform infrared spectroscopy (JASCO Corporation, FT/IR-6100 Tokyo, Japan) with an ATR unit. The UV–Vis absorbance of r-BS was measured using a spectrophotometer (JASCO Corporation, V-770, Tokyo, Japan). The Kubelka–Munk function was used for the Tauc plot.

### Photoelectrochemical property

The r-BS electrode was prepared on a fluorine-doped tin oxide (FTO) glass by a drop-casting method using r-BS powder solution dispersed in ethanol with 5% Nafion™ solution (DE520 CS type, Wako Pure Chemical Industries Ltd., Osaka, Japan). The photocurrent of r-BS was evaluated in an aqueous solution with 0.5 M sodium sulfate (Wako Pure Chemical Industries Ltd., Osaka, Japan) at a pH of 7.0. The working, counter, and reference electrodes were r-BS, Pt plate, and Ag/AgCl, respectively. The r-BS electrode was irradiated with light using a xenon lamp with short-wavelength cutoff filters (cutoffs below 610, 510, 470, 420, 400, and 350 nm). Figure S11a in Supporting Information shows the spectra under these conditions. The photocurrent was recorded using a potentiostat (Hokuto Denko Corp, HZ-7000, Tokyo, Japan) at rest potential under light-on and light-off conditions (light irradiation from 10 to 40 s). The linear sweep voltammogram of the r-BS electrode coated on a carbon substrate was also recorded under chopped light irradiation by a 150 W xenon lamp.

### Hydrogen evolution

The r-BS powder (20 mg) was introduced into a 65 mL glass reactor (see Supporting Information, Fig. S12a), which was cooled using a water-circulating system during light irradiation. Ethanol was added to the reactor as the sacrificial agent. Before irradiation, Ar gas was bubbled through the dispersed r-BS solution. This Ar bubbling procedure was conducted every 12 h after light irradiation using a xenon lamp. The H_2_ concentration was evaluated using a gas chromatograph equipped with a thermal conductive detector and a molecular sieve 5A 60/80 column (SHIMAZU Co. Ltd, GC-2014, Kyoto, Japan). The H_2_ production experiment for calculating the internal quantum efficiency was conducted using a small glass cell (4.5 mL, see Supporting Information, Fig. S12b). The 8 mg r-BS dispersed solution, in which water and ethanol were added, was irradiated by a xenon lamp passed through band-pass filters (490, 450, 420, 390, and 350 nm). Figure S11b shows the spectra of the light irradiation conditions. The formula for calculating the internal quantum efficiency (*IQE*) is described in Note 1 of Supporting Information. The durability test for H_2_ generation was also conducted using a small glass cell (4.5 mL). The 4 mg r-BS dispersed solution, in which water and ethanol were added, was irradiated by a xenon lamp (Light intensity is shown in Fig. S11d).

### Carbon dioxide reduction

A CO_2_ reduction reaction was performed in a small glass cell (4.5 mL). r-BS powder (8 mg) was added to water, and 2,2,2-Nitrilotriethanol (TEOA, Kanto Chemical Co. Inc., Tokyo, Japan) was added to a quartz cell as a sacrificial agent. Before light irradiation, CO_2_ was bubbled through the solution. A xenon lamp was used as the light source, and the CO concentration in the headspace of the reactor was measured using a gas chromatograph equipped with a dielectric barrier discharge ionization detector (Shimadzu, Co. Ltd, GC-BID, GC-2010, Kyoto, Japan). As a control experiment, we performed the same experiment under the Ar bubbling condition instead of the CO_2_ bubbling condition.

An isotope trace experiment was also conducted using CO_2_ with a carbon mass number of 13 (^13^CO_2_). r-BS powder, water, and TEOA were mixed in a small quartz reactor (4.5 mL). After ^13^CO_2_ was bubbled into the mixture for 5 min, the solution was irradiated by a xenon lamp. The evolved ^13^CO was measured by gas chromatography-mass spectroscopy (GC–MS, SHIMAZU Co. Ltd, GCMS-QP2010, Kyoto, Japan).

### Methylene blue decomposition

Before the MB decomposition test, we checked the relationship between the absorbance of MB at 664 nm and MB concentration (calibration curve, Fig. S13), thus, confirming their linear relationship. MB decomposition reaction was conducted in a small glass cell (4.5 mL). First, 4 mg r-BS powder was added to a 0.01 mg/mL MB aqueous solution in the reaction cell. The solution was then irradiated by a Hg-Xe lamp coupled with a UV bandpass color filter (UV-D36B; the spectrum of the irradiated light is shown in Fig. S11c). Two conditions were prepared for the control experiments. First, only the MB solution was irradiated under the same light irradiation conditions to check the photolysis of MB. A mixture of r-BS and MB aqueous solutions was examined in the dark without light irradiation. The MB decomposition rate was evaluated using the change in absorbance at 664 nm, which originated from the MB absorption. For the r-BS-containing sample, the baselines were normalized to cancel the effect of scattering by the r-BS powder.

### Photo-stability test and AFM/KPFM study

The durability test for H_2_ generation was also conducted using a 4.5 mL quartz glass cell (Fig. S12b). The 4 mg r-BS dispersed solution, in which water and ethanol were added, was irradiated by a xenon lamp (Light intensity is shown in Fig. S11d). The morphology of bare r-BS and r-BS after long time irradiation was observed using SEM (JEOL Ltd., JCM-7000, Tokyo, Japan). AFM and KPFM analysis were performed using an AFM Workshop TT-2 equipped with an SPT-20 (Toyo Corporation) surface potential measurement unit. A platinum and iridium (Pt-Ir) coated cantilever (NanoWorld EFM, Pointprobe^®^) was used to perform the tapping mode. A UV LED (365 nm, 4 mW cm^−2^) was employed in the light irradiation experiment. The UV illumination with an incident angle of around 60° and a diameter of 8 mm was set up and focused using a convex lens. In this experiment, an r-BS solution was dropped onto a mica substrate and allowed to dry before the measurement. During the KPFM measurement, a surface potential difference (SPD) takes place due to the work function difference between the tip and sample, and can be expressed as SPD = (*ϕ*_tip_ − *ϕ*_sample_)*e*, where *ϕ*_tip_ and *ϕ*_sample_ are the work functions of the tip and sample, respectively, and *e* is the positive elementary charge. The AFM and KPFM analyses were done using Gwyddion and the cross-sectional profiles were taken as a mean of vertical lines^45^. To evaluate the reduction site in r-BS and to improve the stability of r-BS, platinum (Pt) or rhodium (Rh) nanoparticles were loaded on r-BS particles by a photo-deposition method. Precursors for Pt and Rh were hydrogen hexachloroplatinate(IV) (H_2_PtCl_4_⋅6H_2_O, Kanto Chemical Co., Ltd., Tokyo, Japan) hexahydrate and rhodium(III) chloride trihydrate (RhCl_3_⋅3H_2_O, Wako Pure Chemical Industries Ltd., Osaka, Japan). Ethanol was added into a reactor as a sacrificial agent, and the light was irradiated by a xenon lamp for photo-deposition. After the light irradiation, powder was collected by filtration, washed with Ethanol several times, and dried.

### Supplementary Information

Supplementary Information.

## Data Availability

Data sets generated during the current study are available from the corresponding author on reasonable request.
