# Supplementary material for: Rhombohedral boron monosulfide as a metal-free photocatalyst
Source: Sci Rep. 2023 Nov 9;13:19540. doi: 10.1038/s41598-023-46769-7 (PMC10636132; doi:10.1038/s41598-023-46769-7)
Supplement: Supplementary file 1 — Supplementary Information. [file 41598_2023_46769_MOESM1_ESM.pdf]

Supplementary Information

Rhombohedral boron monosulfide as a metal-free photocatalyst

Keisuke Miyazaki [1], Akira Yamaguchi [1], Haruki Kusaka [2], Norinobu Watanabe [2], Aufandra Cakra Wardhana [1], Satoshi Ishii [3], Akiyasu Yamamoto [4], Masashi Miyakawa [3], Takashi Taniguchi [3], Takahiro Kondo [2,5,6,*], Masahiro Miyauchi [1,*]

1. Department of Materials Science and Engineering, School of Materials and Chemical Technology, Tokyo Institute of Technology, Meguro-ku, Tokyo 152-8552, Japan

2. Department of Materials Science, Institute of Pure and Applied Sciences, University of Tsukuba, Tsukuba 305-8573, Japan

3. Research Center for Materials Nanoarchitectonics, National Institute for Materials Science, Tsukuba 305-0044, Japan

4. Institute of Engineering, Tokyo University of Agriculture and Technology, Tokyo 183-8538, Japan

5. The Advanced Institute for Materials Research, Tohoku University, 2-1-1 Sendai, Miyagi 980-8577, Japan

6. Tsukuba Research Center for Energy Materials Science, Institute of Pure and Applied Sciences and R&D Center for Zero CO_2 Emission Functional Materials, University of Tsukuba, Tsukuba 305-8573, Japan

This file includes:

Figure S1 Schematic illustration for vibration modes of r-BS.

Figure S2 Wide-scanned XPS spectra of r-BS on a graphite tape and of a bare graphite tape.

Figure S3 Linear sweep voltammogram of r-BS electrode under chopping light by a 150 W xenon lamp.

Figure S4 Isotope carbon monoxide (^{13}CO) generation from $^{12}CO_2$ bubbled (a) and $^{13}CO_2$ bubbled (b) conditions under light irradiation.

Figure S5 The absorption spectra of MB dye for before and after light irradiation and those after O_2 bubbling for 20 and 40 mins under dark condition.

Figure S6 The result of durability test for hydrogen production under strong light condition.

Figure S7 XRD patterns and UV-vis spectra of r-BS before and after long time irradiation.

Figure S8 SEM images of as prepared r-BS and long time irradiated r-BS.

Figure S9 XPS spectra for B-1s and S-2p orbitals before and after the photocatalysis test.

Figure S10 Schematic illustration of a capsule for high-pressured r-BS synthesis.

Figure S11 Spectra of light sources which were used for photocatalyst experiments.

Figure S12 (a) A 65 mL reactor with a quartz window for light irradiation. Three septum parts are installed for gas injection and sampling. (b) a 4.5 mL quartz reactor with a septum cap.

Figure S13 Relationship between concentration of methylene blue in aqueous solution and its absorbance at 664 nm.

Note 1. Calculation of the internal quantum yield (IQE).

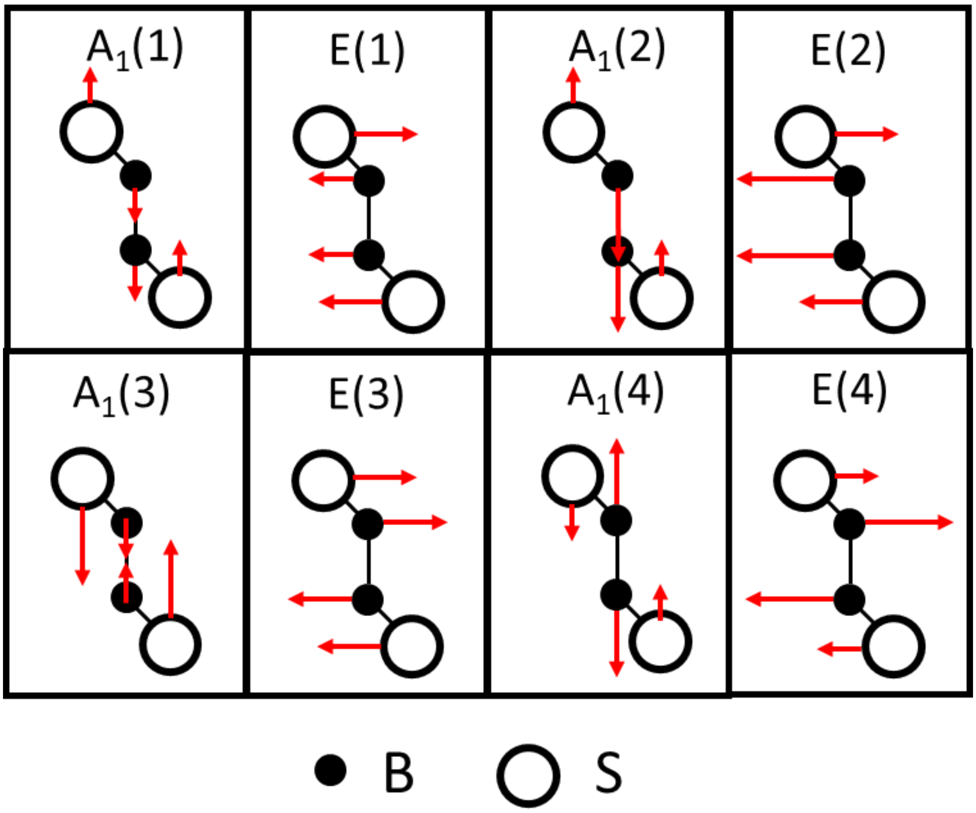

Figure S1 Schematic illustration for vibration modes of r-BS.

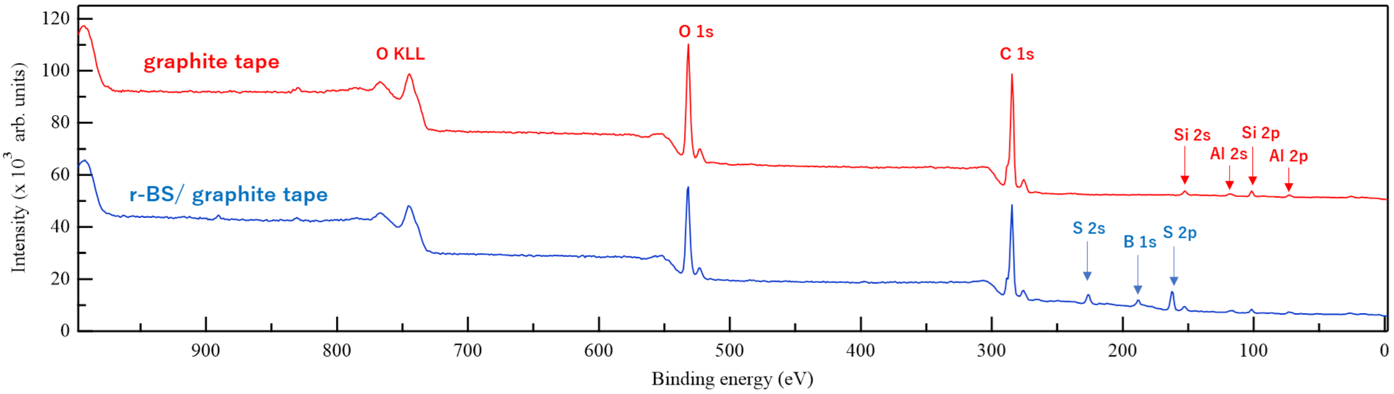

Figure S2 Wide-scanned XPS spectra of r-BS on a graphite tape (blue line) and of a bare graphite tape (red line).

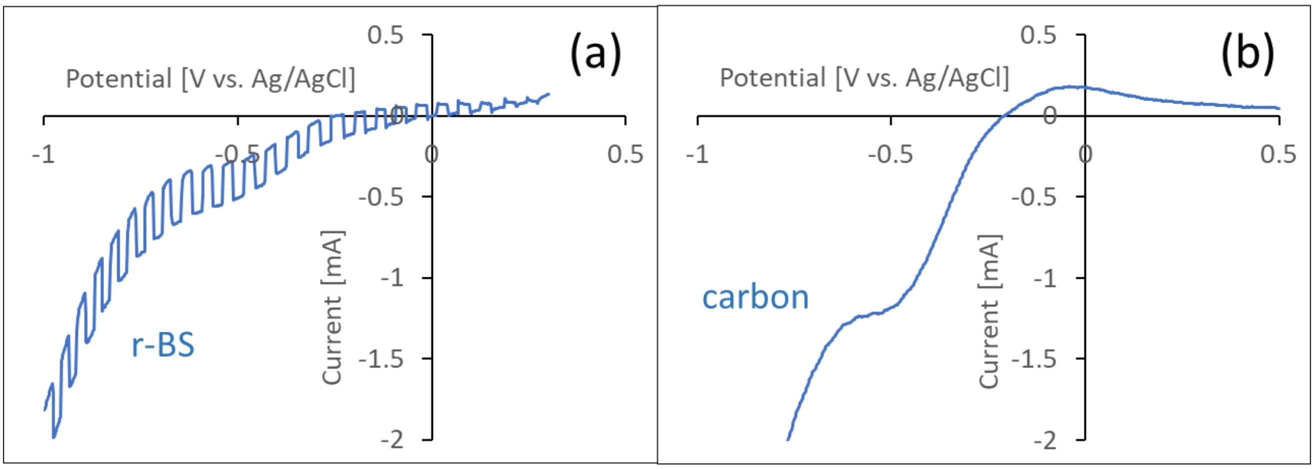

Figure S3 Linear sweep voltammograms of r-BS electrode on a carbon substrate (a) and bare carbon substrate (b) under chopping light by a 150 W xenon lamp.

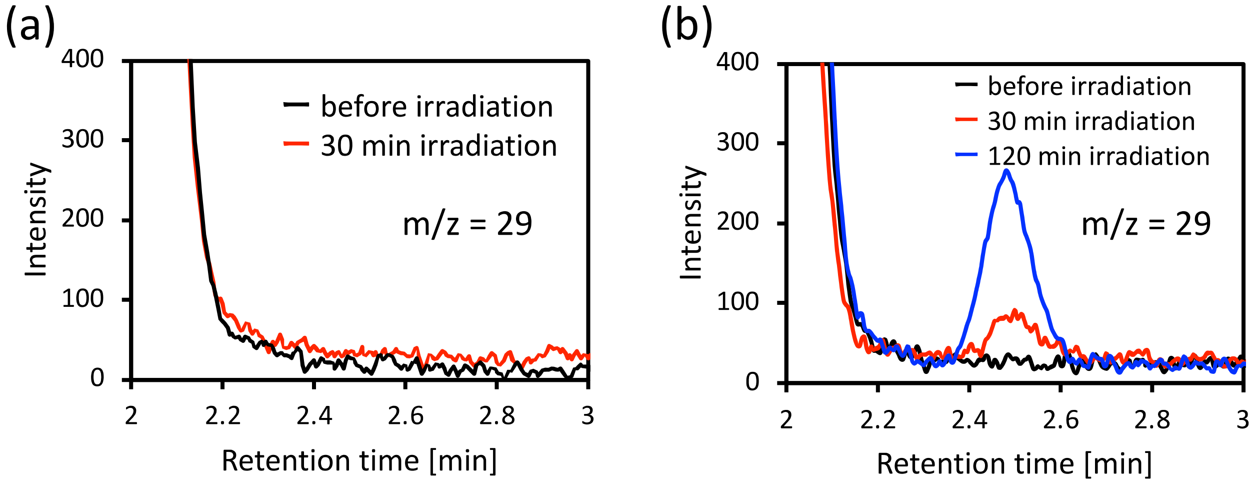

Figure S4 Isotope carbon monoxide (^{13}CO) generation from $^{12}CO_2$ bubbled **(a)** and $^{13}CO_2$ bubbled **(b)** conditions under light irradiation.

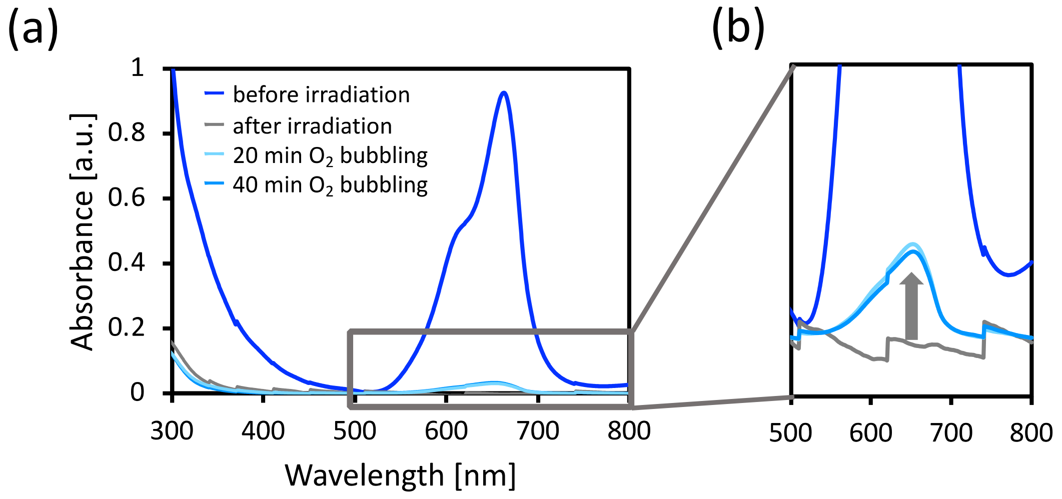

Figure S5 (a) The absorption spectra of MB dye for before and after light irradiation and those after O_2 bubbling for 20 and 40 mins under dark condition. Panel **(b)** is expanded view of the panel (a).

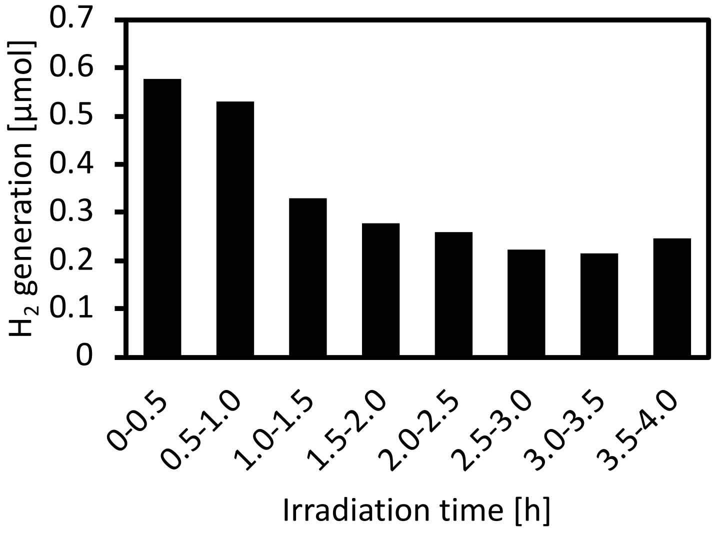

Figure S6 The result of durability test for hydrogen production under strong light condition.

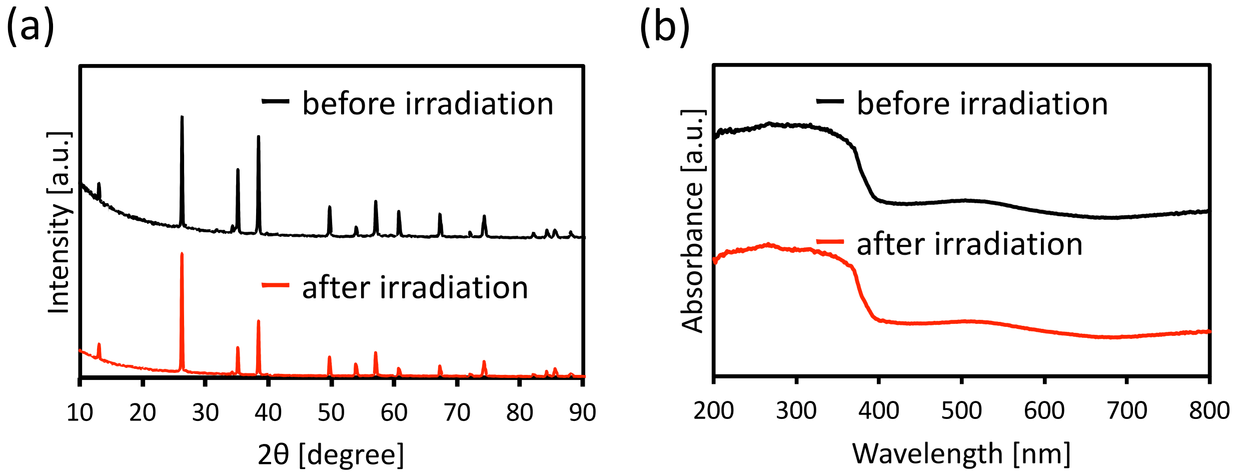

Figure S7 XRD patterns **(a)** and UV-Vis spectra **(b)** of r-BS before and after irradiation.

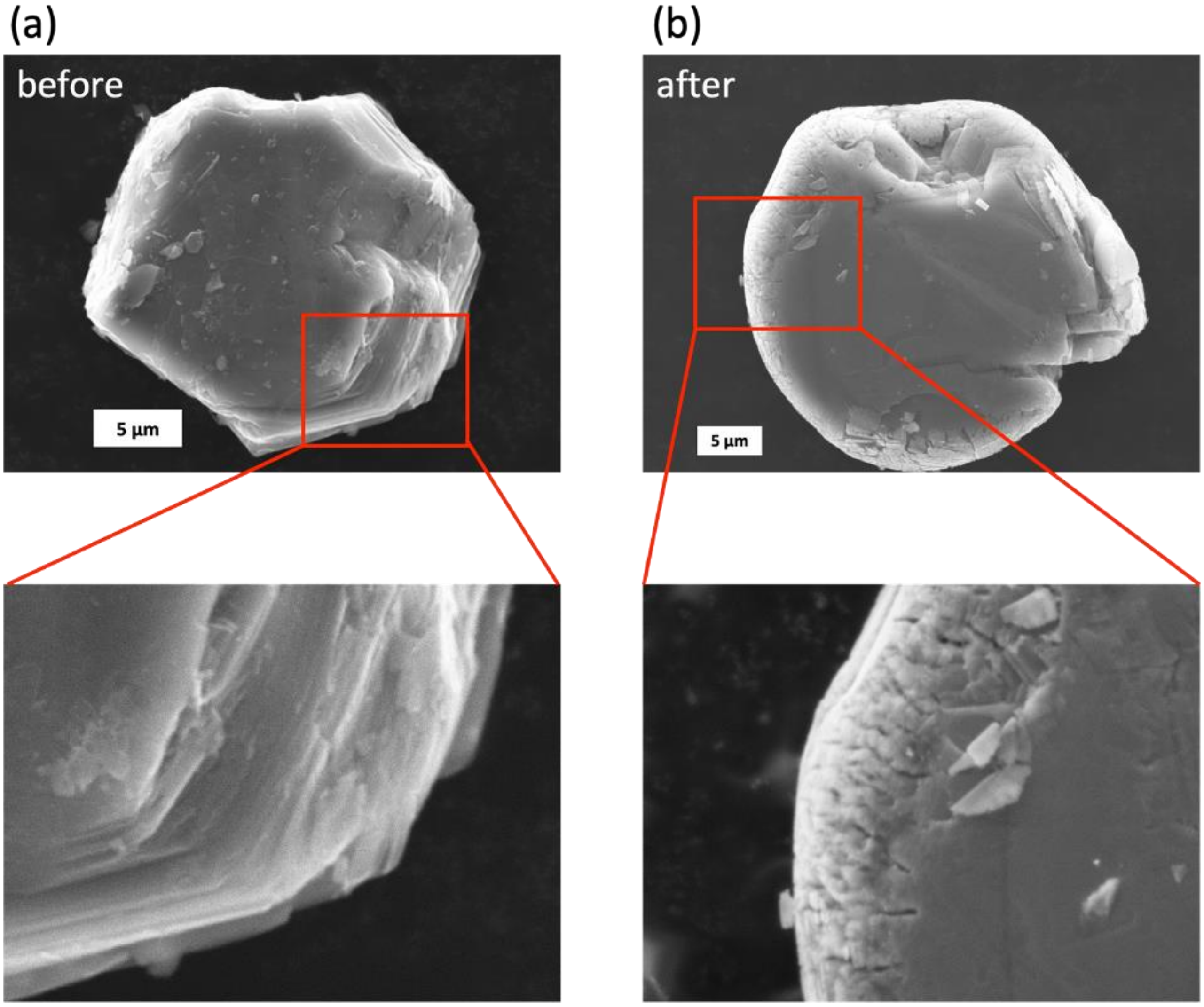

Figure S8 SEM images of as prepared r-BS (a) and long time irradiated r-BS (b).

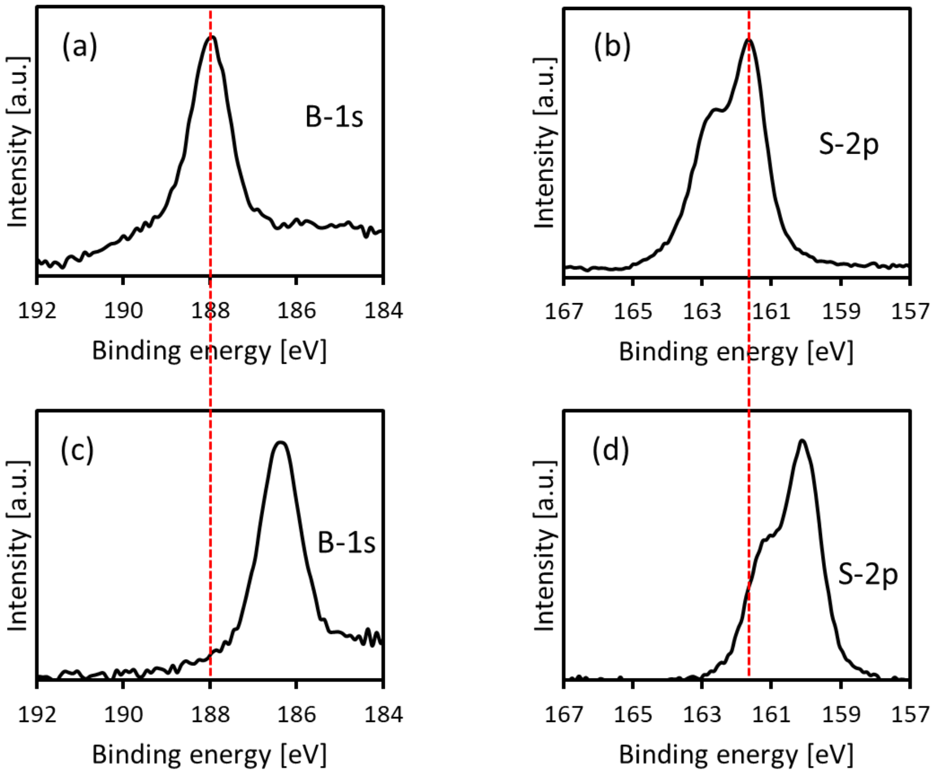

Figure S9 XPS spectra of r-BS for B-1s (a) and S-2p (b) orbitals before the photocatalysis test. Panel (c) and (d) are the results of B-1s and S-2p after the photocatalysis test. As described in the experimental section of the manuscript, the charge build-up effect was calibrated by using the C-1s peak of a graphite tape (284.6 eV). Shifted values for the calibration were -2.5 eV and -2.8 eV for the samples before and after the photocatalysis test, respectively.

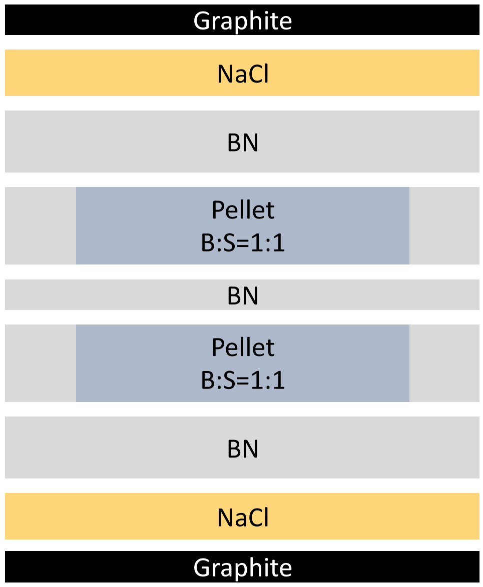

Figure S10 Schematic illustration of a capsule for high-pressured r-BS synthesis.

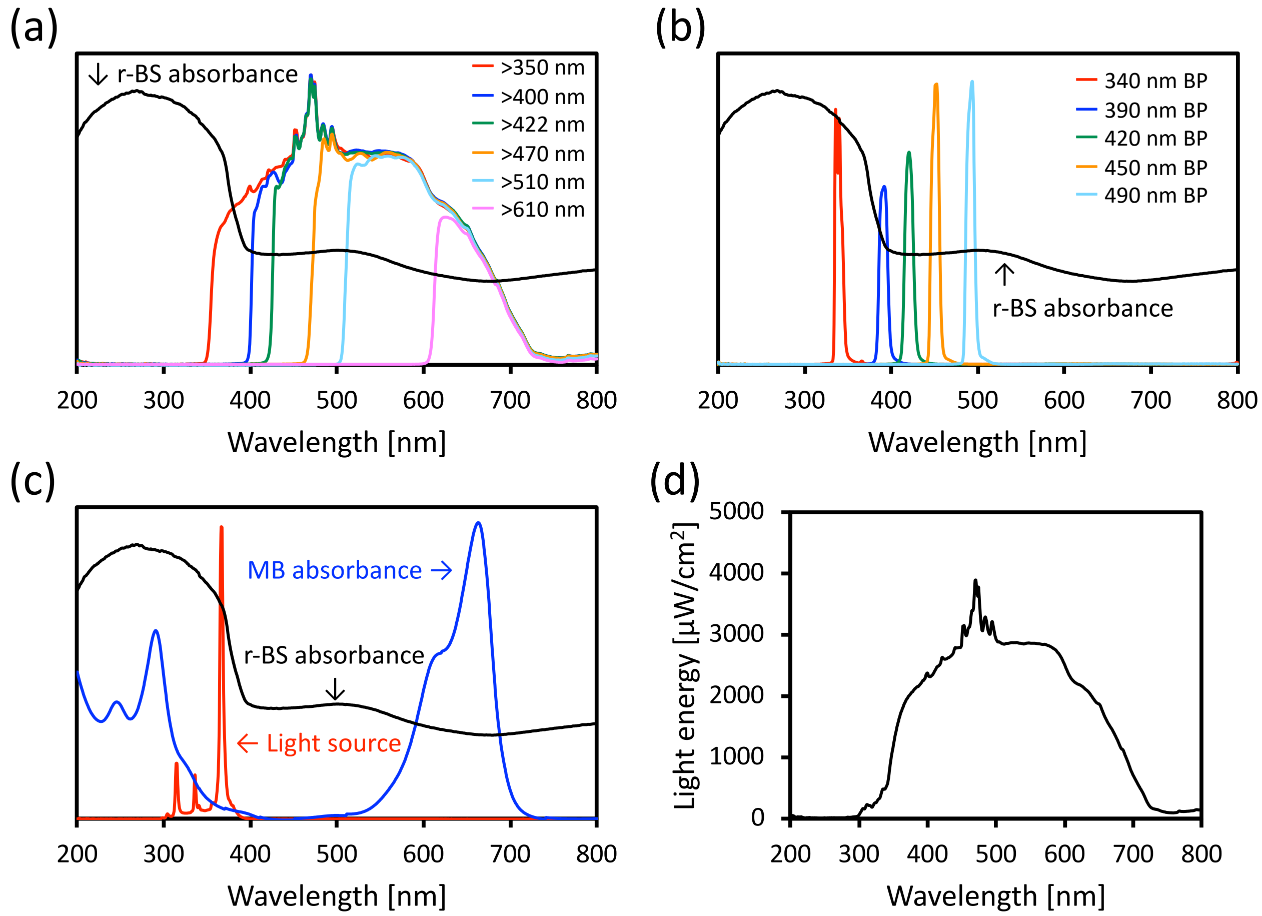

Figure S11 Spectra of light sources which were used for photoelectrochemical experiments **(a)**, hydrogen generation **(b)**, MB decomposition **(c)** and stability test **(d)**.

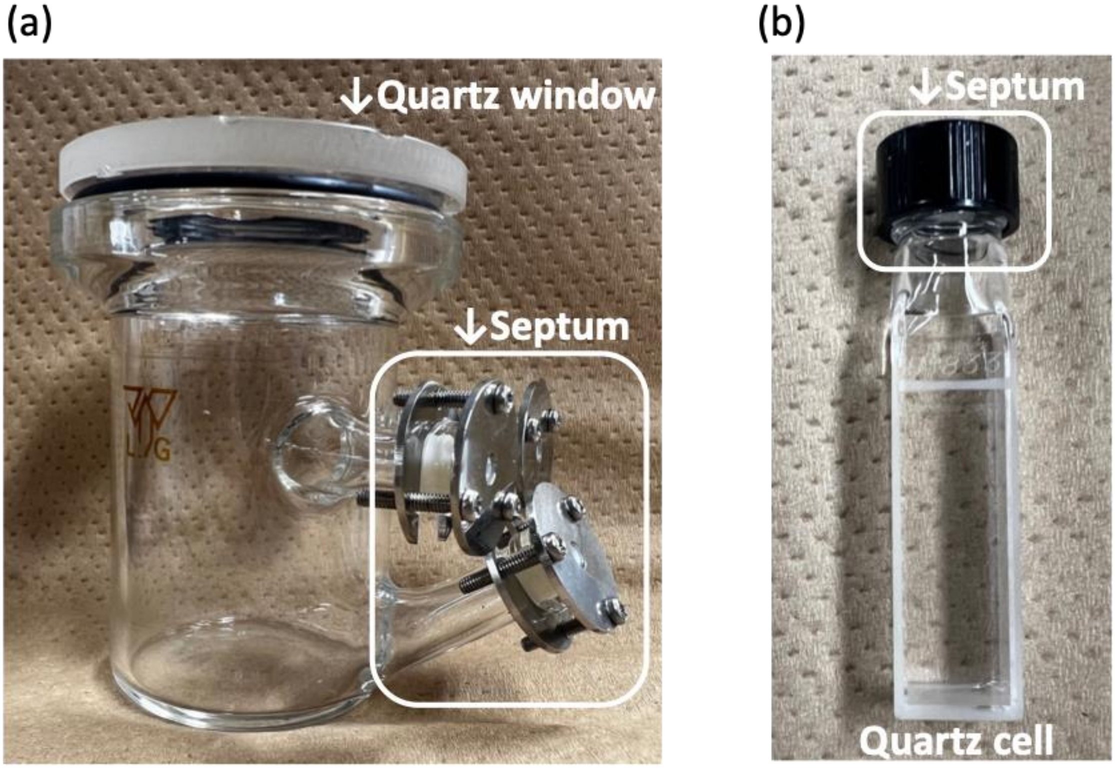

Figure S12 (a) A 65 mL reactor with a quartz window for light irradiation. Three septum parts are installed for gas injection and sampling. **(b)** A 4.5 mL quartz reactor with a septum cap.

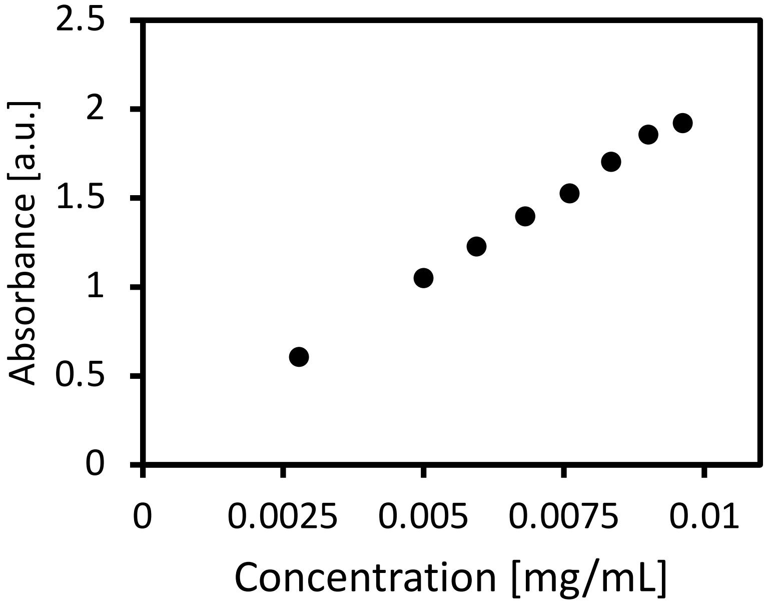

Figure S13 The relationship between concentration of methylene blue in aquaeous solution and its optical absorbance at 664 nm.

Note 1. Calculation of the internal quantum efficiency (*IQE*).

The hydrogen evolution experiment was conducted in small quartz cell. The light source of each wavelength was irradiated to r-BS dispersed in water with the addition of ethanol for 30 min. We calculate the *IQE* from the hydrogen evolution rate determined by the amount of hydrogen production for 30 min divided by absorbed photon numbers determined by the spectrum of the light source and absorption spectrum of r-BS as follows,

$$IQE(x) = \frac{Rate\ of\ used\ electron\ number\ [s^{-1}]}{Absorbed\ photon\ number\ [s^{-1}]} = \frac{H(x)[mol\ s^{-1}] \times 2 \times N_A[mol^{-1}]}{\sum_{\lambda=200}^{\lambda=800}\{(1 - R(\lambda)) \times Photon(\lambda)\}}$$

$$= \frac{H(x)[mol\ s^{-1}] \times 2 \times N_A[mol^{-1}]}{\sum_{\lambda=200\ nm}^{\lambda=800\ nm}\left\{(1 - R(\lambda)) \times \frac{(E(\lambda)[\mu W \cdot cm^{-2}] \times 10^{-6}) \times T[s]}{h[j \cdot s] \times c[m \cdot s^{-1}]/(\lambda[nm] \times 10^{-9})}\right\} \times A[cm^2]}$$

$H(x)[mol\ s^{-1}]$: Hydrogen production rate at diffrednt light source when we usded $x\ nm$ band pass filter

$N_A[mol^{-1}]$: Avogadro number (6.02×10^{23})

$R(\lambda)$: optical reflectance of r − BS powder measured by UV − vis

$E(\lambda)[\mu W \cdot cm^{-2}]$: light energy when the wavelength is λ nm

$T[s]$: Irradiation time$(= 3600)$

$h[j \cdot s]$: Planck constant$(= 6.63 \times 10^{-34})$

$c[m \cdot s^{-1}]$: Speed of light $(= 3.0 \times 10^8)$

$\lambda[nm]$: Wavelength

$A[cm^2]$: Light irradiated area $(= 1.0\ cm^2)$
